# Bridging Personality and Online Prosocial Behavior: The Roles of Empathy, Moral Identity, and Social Self-Efficacy

**DOI:** 10.3389/fpsyg.2020.575053

**Published:** 2020-10-22

**Authors:** Jie Leng, Qingke Guo, Bingqing Ma, Shuyue Zhang, Peng Sun

**Affiliations:** ^1^School of Psychology, Shandong Normal University, Jinan, China; ^2^Department of Psychology, Guangxi Normal University, Guilin, China; ^3^Faculty of Psychology, Beijing Normal University, Beijing, China

**Keywords:** personality, online prosocial behavior, empathy, moral identity, social self-efficacy

## Abstract

Personality has been considered as important influential factors of prosocial behavior (PB). This study aims to investigate whether the personality-PB association revealed in the real world is applicable to cyberspace. Researchers further considered moral identity (MI), empathy, and social self-efficacy as mediators accounting for the association of personality and online prosocial behavior (OPB). Self-reported measures were administrated to 1398 participants from eastern China. Results showed (1) extraversion, agreeableness, conscientiousness, and openness were positively related to OPB, while neuroticism was negatively related to OPB; (2) perspective taking could serve as a mediator between all big five traits and OPB, social self-efficacy did the same job unless the predictor was agreeableness. Empathic concern and MI were less important mediators partly because OPB involves no face-to-face interaction. These findings show that personality has a significant effect on OPB through its influence on moral development.

## Introduction

The association of personality and prosocial behavior (PB) has been examined extensively. Literature shows that agreeableness and extraversion are positively associated with PB (Oda et al., 2014; Habashi et al., 2016), and neuroticism is negatively associated with PB (Habashi et al., 2016; Guo et al., 2018b). Over the past decades, popularization of the Internet facilitated the emergence of a new form of PB, namely online prosocial behavior (OPB). OPB refers to behavior performed voluntarily to help others online without expectation of any reward. Typical OPB includes online donation, online sharing, and online comfort (Sproull, 2011; Erreygers et al., 2018; Zheng et al., 2018). OPB is similar to PB in benefiting others at the cost of the helper (Wright and Li, 2011; Reich, 2017; Guo et al., 2018a). However, the unique characteristics of OPB (e.g., less costly, anonymous, and less social pressured) are noteworthy (Lee and Lee, 2010; Zheng et al., 2018). Personality is an influential factor of online social behaviors (Amichai-Hamburger and Vinitzky, 2010). However, the personality-OPB association has not been well established. Therefore the first aim of this study is to investigate whether the personality-PB association persists in online settings.

If personality is linked to OPB, then what are the mechanisms accounting for this link? Literature suggests that moral cognition and moral emotions are dispositional factors motivating prosocial engagement (e.g., Eisenberg, 2000; Hardy, 2006). Moral identity (MI), the cognitive component of morality, is directly and strongly associated with PB (Blasi, 1980; Aquino and Reed, 2002). Empathy-related responding may be the most frequently studied moral emotions (Eisenberg, 2000). However, from a social cognitive perspective, empathy and MI may not facilitate prosocial engagement if the ability to alleviate others’ distress is absent (Caprara et al., 2012). This suggests that interpersonal self-efficacy beliefs are also important determinants of PB. Based on above theorizing, the second aim of this study is to investigate whether the personality-OPB link can be accounted for by moral cognition (i.e., MI), moral emotion (i.e., empathy), and interpersonal self-efficacy beliefs (i.e., social self-efficacy).

### Personality and OPB

Personality is relatively stable over time and can consistently predict a variety of social behavior (Specht et al., 2011; Hampson, 2012). The relationships between big five personality traits (Costa and McCrae, 1992) and PB have been extensively discussed. Evidence shows that personality is associated with PB assessed through self-report measures, suggesting that agreeableness, extraversion, openness, and conscientiousness are positively associated with PB, while neuroticism is negatively associated with PB (e.g., Oda et al., 2014; Habashi et al., 2016; Xie et al., 2016; Guo et al., 2018b). These findings have been confirmed by researches using behavioral measures in both laboratory settings and in real world (e.g., Ben-Ner and Kramer, 2010; Courbalay et al., 2015; Habashi et al., 2016). However, a recent meta-analysis shows that extraversion and neuroticism are not related to PB measured in economic games (Thielmann et al., 2020). Thus the relationship between personality and PB need to be clearly addressed. To date only a few studies have explored the effects of personality on OPB. For example, openness was found to be positively associated with moral courage and help-giving both in real life and online (Kinnunen et al., 2016); and agreeableness and conscientiousness were positively associated with online information-sharing (Deng et al., 2017). These indicated that extroverted and open-minded (e.g., imaginative, curious) individuals tend to behave more positively online. However, shyness, a primary personality trait locating between introversion and neuroticism, was found to be negatively associated with OPB (Guo et al., 2018a). This citation suggests that introversion and neuroticism may also be negatively associated with OPB. But the effects of personality on OPB have not yet been soundly established, because previous studies have not used a comprehensive measure of OPB (Kinnunen et al., 2016; Deng et al., 2017), or have not directly investigated the roles of big five traits (Guo et al., 2018a).

Though online and offline behavior are similar in many aspects, such as both consume resources of the helpers, have no expectation of return of any benefit, occur in the organized social environment that requires positive social interactions (Amichai-Hamburger, 2008; Wright and Li, 2011; Reich, 2017; Guo et al., 2018a), OPB has its unique features. For instance, helping online is less influenced by the help-seekers’ physical appearance, the helpers can control the extent of involvement and the time schedule (Sproull, 2011). Moreover, OPB occurs anonymously, costs less resources, and generates less social pressures (Lee and Lee, 2010; Zheng et al., 2018). Therefore, whether the relationship between personality and OPB is similar to that in real life is still open to question. Literature shows that social competences developed in offline world are also applicable to online world (Subrahmanyam et al., 2006; Wright and Li, 2011; Reich, 2017). Online and offline worlds are psychologically connected (Wright and Li, 2011), thus the effects of social skills (or deficit) can be extended to the online world. Researchers also found that online behavior is positively related to daily social behavior offline, which indicates the consistence of offline and OPB (Ma et al., 2011; Wright and Li, 2011; Bosancianu et al., 2013). Besides, the cross-situational consistency of personality traits (Sherman et al., 2010; Specht et al., 2011; Hampson, 2012) suggests that personality may be associated with social behaviors in online and offline settings uniformly. Based on this theorizing, we propose that the effect of personality on PB can be extended to OPB.

### The Effects of Empathy, Moral Identity, and Social Self-Efficacy on PB/OPB

Developmental psychologists have divided morality into three dimensions: moral cognition, moral emotion, and moral conduct (e.g., Kochanska et al., 2005; Jennings et al., 2015). MI refers to the extent to which being a moral person is central to that persons’ self-concept (Aquino and Reed, 2002; Hardy and Carlo, 2011). As a social self-schema, MI can be represented by a set of traits organized around self-conception. It is a key psychological mechanism bridging moral reasoning and moral behavior (Aquino and Reed, 2002; Winterich et al., 2009). Aquino and Reed (2002) further distinguished two components (namely implicit and explicit) of MI. The former (MI internalization) reflects directly the degree to which moral traits are important to one’s self-concept, while the latter (MI symbolization) reflects the degree to which an individual is inclined to convey publicly that s/he is a moral person (Aquino and Reed, 2002; Winterich et al., 2013). MI can positively predict charitable behavior (Hardy et al., 2015) and ethical behavior toward organizations (Hertz and Krettenauer, 2016). MI is more strongly predictive of PB than moral judgment, suggesting that MI can bridge the moral judge-action gap (Patrick et al., 2018).

Moral emotions (e.g., guilt, shame, empathy; Tangney et al., 2007) often exert stronger influences on moral actions than moral judgment (Jennings et al., 2015). Empathy refers to understanding and vicariously experiencing others’ emotions (Eisenberg and Miller, 1987; Batson et al., 2015). Empathy plays a fundamental role in moral functioning (Eisenberg, 2000). According to Davis (1983), empathy contains two cognitive components, namely perspective taking (PT, spontaneously understanding other people’s point of views) and fantasy (FS, imaginatively understanding the feelings of fictional characters in books or movies), and two emotional components, namely empathic concern (EC, an other-oriented feeling of sympathy or concern for the misfortune of others) and personal distress (PD, a self-oriented feeling of discomfort and uneasy when witnessing others in need). Higher scores on empathy indicate better abilities in understanding and experiencing other people’s mental states, and greater sensitivity to their needs (Masten et al., 2011). Other-oriented empathic responses (e.g., EC) elicit behaviors aiming at reducing the distress of the victims, thus are more strongly associated with prosocial engagement. However, self-oriented responses (e.g., PD) are more likely to reduce uncomfortable feelings of the witness, leading to avoidance responses if able to do so (Eisenberger and Fabes, 1990; Carlo et al., 1999; Klimecki and Singer, 2011; Habashi et al., 2016). Empathy has also been shown to facilitate OPB. In cyberspace, empathic individuals show greater willingness to share, help, and donate (Khang and Jeong, 2016; Farrelly and Bennett, 2018).

Social self-efficacy may be associated with moral self-strength that can directly motivate moral actions (Jennings et al., 2015; Khang and Jeong, 2016). It refers to confidence in one’s ability to participate in social interactions (Smith and Betz, 2000; Alessandri et al., 2009). People who lack social self-efficacy usually are unconfident in dealing with interpersonal interactions and solving problems for the victim, thereby showing less prosociality (Bandura, 1997; Habashi et al., 2016). Interpersonal self-efficacy influences the efficiency in managing social relationships and engaging in other people’s emotional experiences, thereby exerting a direct impact on PB (Caprara and Steca, 2005). Caprara et al. (2010) found that trait interpersonal self-efficacy contributed to prosocial engagement. Recent literature suggests that the social self-efficacy and PB association is also applicable to online settings, showing that the belief in the ability to manage online social relationships positively predict OPB (Khang and Jeong, 2016).

### Personality and MI, Empathy, and Social Self-Efficacy

A growing body of literature has examined the association between personality and moral behaviors, such as voluntary helping, cooperation, fairness, inclusiveness/prejudice, and universalism (for a review, see Smillie et al., 2019). This suggests that personality is predictive of moral cognition, emotion, and conduct.

Agreeableness and conscientiousness have been considered as quasi-moral traits that contribute to moral behaviors (Miller, 2007). For example, Krettenauer et al. (2014) found that agreeableness and conscientiousness can positively predict moral decision-making in hypothetical moral dilemmas and subsequent moral emotions. Agreeableness and conscientiousness have been considered as indicators of moral character that are inversely predictive of deviant behaviors (Kim and Cohen, 2015), such as academic dishonesty (Williams et al., 2010). These findings suggest that they can facilitate MI development. Openness is also associated with moral functioning. It is related to emotional sensitivity, social tolerance, political liberalism, and universalism (Miller, 2007). Ferguson et al. (2019) found that openness may facilitate costless prosociality that maximizing others’ payoffs. Extraversion and neuroticism have seldom been studied as quasi-moral traits (Miller, 2007), suggesting that MI cannot account for their associations with PB.

All dimensions of the Big Five may be connected with empathy. Mooradian et al. (2011) found that agreeableness, extraversion, and conscientiousness were significantly correlated with PT and EC; neuroticism was correlated with PT (inversely) and PD, openness was correlated with all components of empathy. Highly agreeable people, featured by kindness and understanding, tend to have higher levels of PT and EC (Barrio et al., 2004; Melchers et al., 2016; Song and Shi, 2017). Neurotic individuals are susceptible to situational social stressor (Lee, 2009). They tend to be overwhelmed by negative emotions (PD) in front of the victims (Barrick and Mount, 1991; Roccas et al., 2002; Mooradian et al., 2011). Open-minded people are curious and imaginative (Barrick and Mount, 1991; Roccas et al., 2002), thus they have better PT and FS abilities. Conscientious people tend to manage social interactions (e.g., other people’s emotions and desires) responsibly (Barrick and Mount, 1991; Miller, 2007; Hampson, 2012), therefore higher levels of empathy (e.g., PT, EC) are also expected in them. Extraversion is characterized by sociability and positive emotionality (Hampson, 2012). Greater responsiveness to others’ emotions (e.g., PT, EC) is expected in extroverted individuals because positive emotions can broaden their scope of attention in social settings thereby triggering more positive responses (Fredrickson, 2004).

The association between personality and social self-efficacy has also been examined in previous literature. Mak and Tran (2001) found that extraversion (featured by sociability and positive emotionality), openness (featured by open-mindedness and tolerance for ambiguity), conscientiousness (featured by persistence, carefulness, and effortful control) are contributive to successful interpersonal communications, leading to higher levels of social self-efficacy in intercultural situations. Neuroticism (featured by social anxiety and avoidance in social situations), in contrast, was inversely associated with social self-efficacy (Mak and Tran, 2001).

### This Study

The co-construction theory (Subrahmanyam et al., 2006) and the rich-get-richer/poor-get-poorer hypothesis (Kraut et al., 2002) both indicate that off-line and online world are connected. Based on these findings, we propose that people’s social preferences in real world can extend to online world. For example, social skills deficit may hinder social interactions in both online and offline settings (Kraut et al., 2002; Wright and Li, 2011; Reich, 2017). And people who have a prosocial personality (Habashi et al., 2016) are more likely to help the needy regardless they emerge in real life or in cyberspace (Wright and Li, 2011; Guo et al., 2018a). Given above, we assume that agreeableness (*Hypothesis 1a*), extraversion (*Hypothesis 1b*), conscientiousness (*Hypothesis 1c*), and openness (*Hypothesis 1d*) were positively associated with OPB, while neuroticism (*Hypothesis 1e*) was negatively associated with OPB.

The mechanisms through which personality gets outside the skin (Hampson, 2012) to exert influences on PB have scarcely been investigated. MI, empathy, and social self-efficacy play fundamental roles in motivating prosocial engagement (Eisenberg, 2000; Aquino and Reed, 2002; Jennings et al., 2015). The similarity of PB and OPB suggests that MI, empathy and social self-efficacy may mediate the personality-OPB association. Specifically, we assume that extraversion may be positively associated with OPB via empathy and social self-efficacy (*Hypothesis 2a*); agreeableness may be positively associated with OPB (Eisenberg, 2000; Miller, 2007; Kim and Cohen, 2015) via empathy and MI (*Hypothesis 2b*); conscientiousness may be positively associated with OPB (Eisenberg, 2000; Miller, 2007; Kim and Cohen, 2015) via empathy, MI and social self-efficacy (*Hypothesis 2c*), and openness may do the same job (*Hypothesis 2d*); neuroticism may be negatively associated with OPB via empathy and social self-efficacy (*Hypothesis 2e*).

In this study only the internalization aspect of MI was examined (cf. Aquino et al., 2009; Decelles et al., 2012) because it directly taps the essence of this construct. Existing literature suggests that the internalization aspect of MI is more strongly predictive of PB than the symbolization aspect (Winterich et al., 2013). Four dimensions of empathy were all examined in this study because they play different roles in influencing PB (Habashi et al., 2016).

## Materials and Methods

### Participants and Procedure

A total of 1398 college students (M_*age*_ = 19.04, SD_*age*_ = 1.22, *N*_*male*_ = 566) from eastern China participated in this study in order to obtain course credits. 48.3% of their fathers and 60.2% of their mothers obtained a junior high school degree or lower, 28.0% of their fathers and 22.7% of their mothers obtained a senior high school degree, and 23.7% of their fathers and 17.1% of their mothers obtained a college degree. About 26.3% of them had a per capita monthly income of ¥500–1500, 36.8% had ¥1500–3000, and 36.8% had more than ¥3000.

Informed consents were obtained from them. They completed a suite of questionnaire items measuring their demographic characteristics, big five personality, OPB, empathy, social self-efficacy, and MI (totally 107 items). Each time these measures were group-administrated to 30–50 participants in university classrooms, with the supervision of two trained research assistants. Each participant received a pen as a gift (costing ¥3) in compensation for participation and was informed of the purpose of the study. This study was in accordance with the ethical standards of the academic committee at Shandong Normal University and the 1964 Helsinki declaration and its later amendments. There were 63 participants whose responses had not been entered into the research database due to invalid answers (giving the same answer to all items of a questionnaire or failing to answer at least one of the questionnaire items).

### Measures

#### Personality

The Big Five Inventory (BFI-44; Benet-Martinez and John, 1998) includes 44 items. Each item is rated using a five-point scale ranging from 1 (strongly disagree) to 5 (strongly agree). Example items are “Can be tense” (neuroticism; eight items), “Is talkative” (extraversion; eight items), “Has an active imagination” (openness; ten items), “Has a forgiving nature” (agreeableness; nine items), and “Does a thorough job” (conscientiousness; nine items). BFI has been validated in different cultural backgrounds (Guo et al., 2018a; Oda et al., 2014). In this study, McDonald’s omega (Dunn et al., 2014; Hayes and Coutts, 2020) of five sub-scales’ were 0.74, 0.77, 0.72, 0.71, and 0.76, respectively. Total score of each dimension was taken. Confirmatory factor analysis (CFA) showed that BFI-44 has good factorial validity (χ^2^/df = 2.48; CFI = 0.94; TLI = 0.92; RMSEA = 0.03).

#### Online Prosocial Behavior

Online prosocial behavior was measured by a short version of the Internet Altruistic Behavior Scale (IABS) (Zheng et al., 2011). It contains fourteen four-point (1 = never, 4 = always) items, measuring three dimensions of OPB (i.e., online support, online mentoring, online sharing). Sample items are “Caring and encouraging others” (online support), “Guiding others how to use the Internet more efficiently”(online mentoring), and “Sharing with others experiences of successful learning” (online sharing). Total score of all items represents the levels of OPB. CFA of the 14-item IABS yielded satisfying goodness of fit statistics (χ^2^/df = 10.96; CFI = 0.91; TLI = 0.90; RMSEA = 0.08). McDonald’s omega was 0.90.

#### Empathy

The Chinese version (Siu and Shek, 2005) of the Interpersonal Reactivity Index (IRI-C) (Davis, 1983) was used to assess levels of empathy. This measure consists of 22 items measuring four dimensions (i.e., PT, EC, FS, and PD) of empathy. Each item was rated using a five-point scale ranging from 0 (does not describe me well) to 4 (describes me well). Example items are “Describe self as soft hearted” (EC; six items), “Tend to loss control in emergences” (PD; five items), “Imagine how people feel before I criticize them” (PT; five items), “Felt like one of the character in play/movie” (FS; six items). CFA was conducted to assess construct validity of this scale, yielding acceptable goodness of fit indices (χ^2^/df = 3.96; CFI = 0.93; TLI = 0.90; RMSEA = 0.05). McDonald’s omega for PT was 0.71, for EC was 0.59, for FS was 0.64, and for PD was 0.75. Total score of each dimension was taken.

#### Moral Identity

Chinese version of MI measure (MIM-C) was developed by Wan and Yang (2008) based on Aquino and Reed (2002). Participants were asked to read nine traits (compassionate, caring, friendly, fair, helpful, honest, generous, hardworking, and kind) describing a person, then imagine a person who has these characteristics and visualize how this person think, feel, and behave. Then the internalization subscale (including nine five-point Likert-type items) was used to assess MI. A sample item is “Being a person who has these characteristics makes me feel good (1 = strongly disagree, 5 = strongly agree).” Total score of all items was taken. McDonald’s omega was 0.95. Satisfying construct validity was found by CFA (χ^2^/df = 5.73; CFI = 0.99; TLI = 0.99; RMSEA = 0.06).

#### Social Self-Efficacy

Chinese version (Fan et al., 2006) of the Scale of the Perceived Social Self-Efficacy (PSSE) (Smith and Betz, 2000) contains 18 five-point items (1 = no confidence at all, 5 = complete confidence), assessing perceived self-confidence in social interactions. An example item is “How confident are you in making friends?” CFA of the scale yielded satisfying goodness of fit statistics (χ^2^/df = 7.25; CFI = 0.93; TLI = 0.91; RMSEA = 0.07). McDonald’s omega was 0.91. Total score of all items was taken to represent level of social self-efficacy.

## Results

Procedural and statistical remedies were both applied to control common method bias (Podsakoff and Organ, 1986). First, participants were informed that they should complete the items according to their real experiences, and the results were irrelevant to their school records and would be kept confidentially. Second, Harman’s one-factor analysis was conducted to examine whether the first factor can account for a large amount of variance. Exploratory factor analysis of all questionnaire items yielded 20 factors (communality was 0.58) with eigenvalue over 1, with the first factor only explaining 14.96% of the total variance. This suggests that common method bias was not serious.

### Correlation Analysis

Descriptive statistics and partial correlation coefficients (control variables were gender, monthly income, and father’ and mother’ educational level) were presented in Table 1. Results indicated that correlations among research variables mostly reached statistical significance. Five personality traits except neuroticism were positively related to OPB, PT, EC, social self-efficacy, and MI internalization. Neuroticism was positively associated with PD, while other personality dimensions’ were negatively associated with PD. OPB was positively correlated with PT, EC, FS, social self-efficacy, and MI internalization.

**TABLE 1:** Partial correlation coefficients between variables (*N* = 1398).

	**M (SD)**	**1**	**2**	**3**	**4**	**5**	**6**	**7**	**8**	**9**	**10**	**11**	**12**
1 extraversion	25.28 (5.10)	1											
2 agreeableness	33.31 (4.75)	0.33***	1										
3 conscientiousness	29.03 (5.07)	0.31***	0.39***	1									
4 openness	35.85 (5.85)	0.39***	0.30***	0.36***	1								
5 neuroticism	23.75 (4.83)	−0.49***	−0.45***	−0.39***	−0.22***	1							
6 PT	13.42 (3.19)	0.17***	0.25***	0.25***	0.35***	−0.15***	1						
7 FS	16.52 (3.94)	0.09**	0.17***	0.06*	0.20***	0.07*	0.32***	1					
8 EC	16.00 (3.51)	0.11***	0.36***	0.18***	0.14***	−0.08**	0.28***	0.40***	1				
9 PD	10.72 (3.78)	−0.21***	−0.16***	−0.26***	−0.11***	0.47***	0.05	0.14***	−0.02	1			
10 social self-efficacy	60.60 (10.35)	0.54***	0.34***	0.32***	0.40***	−0.40***	0.29***	0.15***	0.18***	−0.28***	1		
11 moral identity internalization	26.98 (10.63)	0.15***	0.38***	0.44***	0.22***	−0.06*	0.28***	0.42***	0.83***	0.21***	0.20***	1	
12 OPB	33.02 (8.42)	0.25***	0.25***	0.22***	0.30***	−0.20***	0.27***	0.14***	0.17***	−0.04	0.34***	0.19***	1

*p < 0.05; **p < 0.01; ***p < 0.001.

### Multiple Mediation Analysis

In order to identify multiple mediation mechanisms in the relationship between personality and OPB, structural equation models (SEM) were constructed using Amos (Version 20; Preacher and Hayes, 2008). Each time one personality trait was used as an independent variable and OPB was used as the outcome, with different mediators accounting for their association. Besides, demographic variables (i.e., gender, monthly income, father’s and mother’s educational level) was used as control variables in each model (see Model 1 to 5). All hypothesized models fitted the data well (Table 2). Multiple mediation analysis was conducted using PROCESS micro for SPSS (Hayes, 2012) with bias-corrected bootstrap method (5000 bootstrap samples, 95% confidence interval). Results were presented in Table 3.

**TABLE 2:** Model fit indexes.

**Model**	**χ2/df**	**CFI**	**TLI**	**RMSEA**
Model 1 (extraversion)	4.505	0.960	0.925	0.050
Model 2 (agreeableness)	5.281	0.969	0.941	0.055
Model 3 (conscientiousness)	6.409	0.964	0.944	0.062
Model 4 (openness)	6.415	0.959	0.921	0.062
Model 5 (neuroticism)	3.137	0.979	0.955	0.039

**TABLE 3:** Standardized specific indirect effect of personality on online prosocial behavior.

**Variables**	**Direct effect**	**Indirect effects**	**95% Bootstrap CI**	
			**Bootstrap LLCL**	**Bootstrap ULCL**
**Extraversion**	0.160		0.065	0.255
→perspective taking		0.042	0.025	0.064
→empathic concern		0.013	0.004	0.029
→fantasy		0.002	−0.006	0.013
→personal distress		−0.014	−0.035	0.005
→social self-efficacy		0.218	0.151	0.285
(total)		0.261	0.210	0.345
**Agreeableness**	0.282		0.182	0.381
→perspective taking		0.089	0.061	0.123
→empathic concern		−0.038	−0.105	0.029
→fantasy		0.009	−0.008	0.027
→personal distress		0.016	−0.001	0.038
→moral identity internalization		0.082	0.013	0.156
(total)		0.158	0.107	0.213
**Conscientiousness**	0.215		0.090	0.340
→perspective taking		0.053	0.031	0.080
→empathic concern		0.038	0.003	0.080
→fantasy		0.002	−0.002	0.012
→personal distress		−0.035	−0.070	−0.007
→moral identity internalization		−0.064	−0.167	0.038
→social self-efficacy		0.146	0.109	0.192
(total)		0.141	0.037	0.244
**Openness**	0.257		0.165	0.349
→perspective taking		0.068	0.037	0.101
→empathic concern		0.011	−0.013	0.139
→fantasy		−0.000	−0.020	0.019
→personal distress		−0.006	−0.021	0.004
→moral identity internalization		0.012	−0.026	0.052
→social self-efficacy		0.161	0.115	0.214
(total)		0.246	0.192	0.305
**Neuroticism**	−0.179		−0.281	−0.078
→perspective taking		−0.038	−0.061	−0.021
→empathic concern		−0.009	−0.024	−0.002
→fantasy		0.003	−0.003	0.013
→personal distress		0.059	0.011	0.109
→social self-efficacy		−0.186	−0.241	−0.138
(total)		−0.172	−0.250	−0.138

*Note: Five personality dimensions’ effects on online prosocial behavior through multiple mediation mechanisms were present in this table, respectively. The bold words stands for independent variables and the dependent variable of the five models is online prosocial behavior. (total) represents the total indirect effect. Gender, Monthly income, and father’ and mother’ educational level served as control variables.*

**FIGURE 1:**
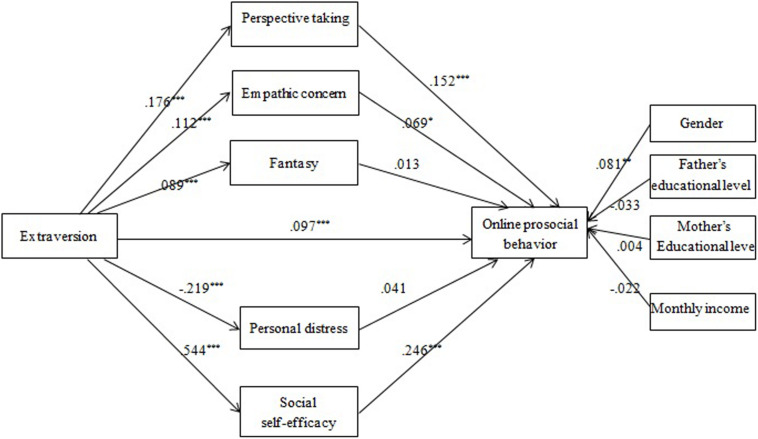
The effects of multiple mediators in the extroversion-OPB association (Model 1): Coefficients standardized. *p < 0.05; **p < 0.01; ***p < 0.001.

Empathy and social self-efficacy were hypothesized to be mediators in the extraversion-OPB association (Model 1). Mediation analysis showed that PT, EC, FS, PD, and social self-efficacy could be significantly predicted by extraversion, but only PT, EC, and social self-efficacy had significant positive effects on OPB (Figure 1). The mediating effect of PT, EC, and social self-efficacy were significantly positive, explaining 10, 3, and 52% of the total effect, respectively.

**FIGURE 2:**
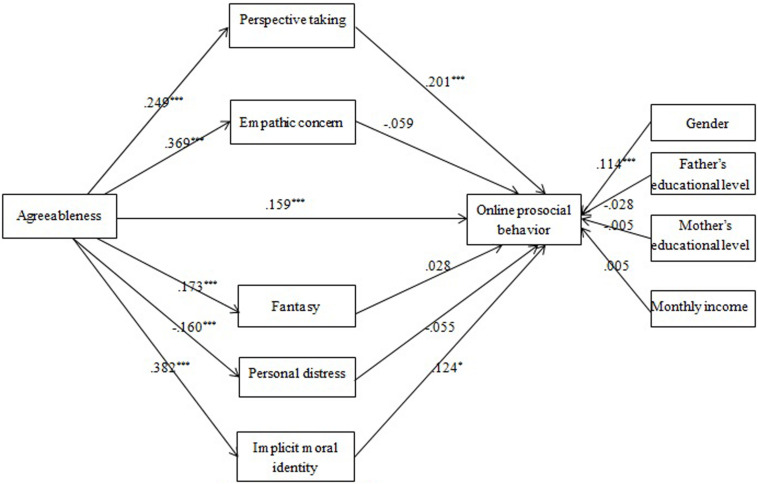
The effects of multiple mediators in the agreeableness-OPB association (Model 2): Coefficients standardized. *p < 0.05; ***p < 0.001.

Empathy and MI internalization were assumed to mediate the agreeableness-OPB association (Model 2). Mediation analysis showed that PT, EC, FS, PD, and MI internalization could be significantly predicted by agreeableness, but only PT, MI internalization positively predicted OPB (Figure 2). Thus the mediating roles of PT and MI internalization were identified, explaining 20 and 19% of the total effect, respectively.

**FIGURE 3:**
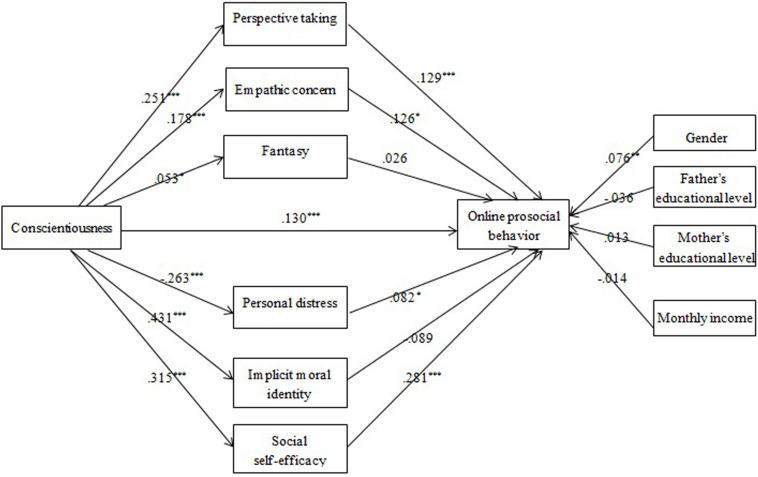
The effects of multiple mediators in the conscientiousness-OPB association (Model 3): Coefficients standardized. *p < 0.05; **p < 0.01; ***p < 0.001.

Empathy, MI internalization, and social self-efficacy were assumed to mediate the conscientiousness-OPB association (Model 3). Mediation analysis (Figure 3) showed that all hypothesized mediators could be significantly predicted by conscientiousness. However, only PT, EC, PD, and social self-efficacy had significantly positive effects on OPB. Thus the mediating roles of PT, EC, PD, and social self-efficacy were identified, explaining 15, 11, 10, and 41% of the total effect, respectively.

**FIGURE 4:**
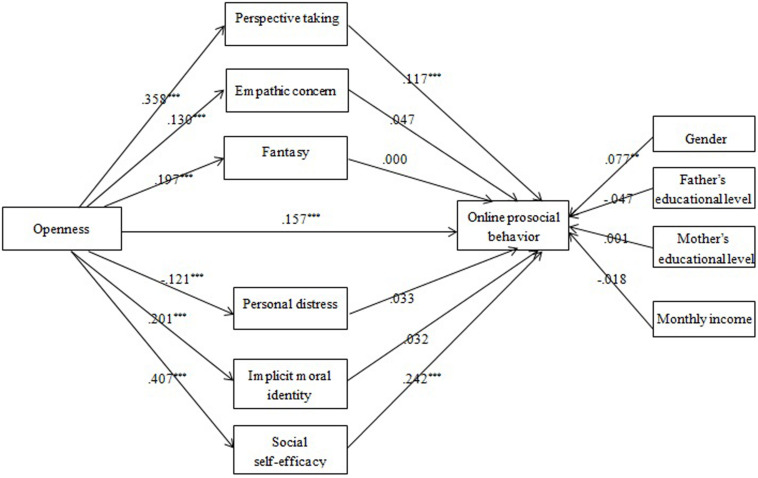
The effects of multiple mediators in the openness-OPB association (Model 4): Coefficients standardized. **p < 0.01; ***p < 0.001.

Empathy, MI internalization, and social self-efficacy were assumed to mediate the openness-OPB association (Model 4). Mediation analysis showed that openness had significant effects on PT, EC, FS, PD, MI internalization, and social self-efficacy. Among them, PT and social self-efficacy had a significantly positive effect on OPB (Figure 4). Thus the mediating roles of PT and social self-efficacy were identified, explaining 13 and 32% of the total effect, respectively.

**FIGURE 5:**
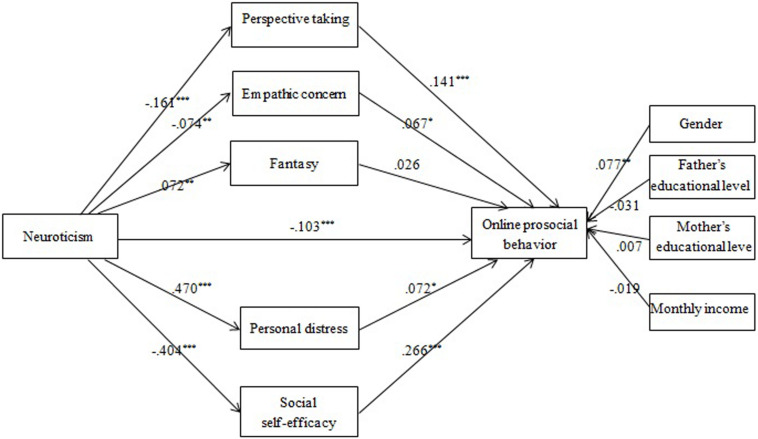
The effects of multiple mediators in the neuroticism-OPB association (Model 5): Coefficients standardized. *p < 0.05; **p < 0.01; ***p < 0.001.

Empathy and social self-efficacy were assumed to mediate the neuroticism-OPB association (Model 5). As shown in Figure 5, PT, EC, FS, PD, and social self-efficacy could be significantly predicted by neuroticism, in turn PT, EC, PD, and social self-efficacy positively predicted OPB. Thus the mediating roles of PT, EC, PD, and social self-efficacy were identified, explaining 11, 3, 17, and 53% of the total effect, respectively.

## Discussion

Consistent with *Hypothesis 1a–e*, we found that the effects of extraversion, agreeableness, conscientiousness, and openness on OPB were all positively significant, while the effect of neuroticism was negatively significant. Our findings support the co-construction theory (Subrahmanyam et al., 2006) and the rich-get-richer/poor-get-poorer hypothesis (Kraut et al., 2002). That is, the effects of personality on social behaviors also apply to cyberspace (Oda et al., 2014; Habashi et al., 2016; Guo et al., 2018a). Though there are many differences between PB and OPB (Sproull, 2011), their associations with personality are consistent. For example, a negative neuroticism-OPB association suggested that the negative effects of low self-efficacy and lack of social skills on positive online social behavior cannot be reduced by convenience and anonymity of the Internet.

### Potential Mediators in the Personality-OPB Link

From the multiple mediation models, we can learn that the relationships between five personality traits and OPB are all partly explained by several assumed moral-related mediators, especially PT and social self-efficacy.

In consistent with *Hypothesis 2a*, we found that extraversion could influence OPB through PT, EC, and social self-efficacy. A fundamental feature of extraversion is positive emotionality (Hampson, 2012). According to Fredrickson (2004), positive emotions can expand the scope of cognition and build enduring social resources, leading to greater engagement with the environments. Extended cognitive and emotional resources may lead an extrovert individual to be capable of caring for others’ feelings (EC) and thinking from others’ perspectives (PT) (Rosen and Kluemper, 2008). Melchers et al. (2016) also found that extraversion was positively related to PT and EC. Further, PT and EC facilitate approaching responses to others’ sufferings, which in turn increase PB/OPB (Guo et al., 2018a). Moreover, extraverted individuals are sociable and talkative (Barrick and Mount, 1991), they have high self-efficacy in dealing with interpersonal relationships and engage in social interactions (Di Giunta et al., 2010). This belief contributes not only to positive social behaviors in real life (Caprara and Steca, 2005; Falanga et al., 2014), but also to social engagement in cyberspace (Khang and Jeong, 2016).

In consistent with *Hypothesis 2b*, we found that agreeableness could increase OPB via higher levels of PT and MI internalization. Agreeableness contains some traditional virtues such as kindness, cooperation, and soft-heartedness (Barrick and Mount, 1991), and has been viewed as a quasi-moral traits (Miller, 2007). It is reasonable that agreeable people can develop a higher level of MI which in turn increases their engagement in moral behavior like PB/OPB (Winterich et al., 2013). Furthermore, agreeable individuals are better in adopting other’s views and understanding other people’s needs (Song and Shi, 2017), and these PT skills are better predictors of OPB. Additionally, we found that unlike findings in real life situations, EC did not mediated the agreeableness-OPB association. Einolf (2008) proposed that the relationship between EC and helping are weak when help-seekers are not at the scene. OPB does not involve face-to-face interactions between the help-seekers and helpers (Sproull, 2011), thus the perception of the help-seekers’ emotional states may be inaccurate (Barrett et al., 2011). Konrath et al. (2011) also suggested that no face-to-face interactions in cyberspace affect the perception and response to the suffering of help-seekers.

Partly in consistent with *Hypothesis 2c*, the effect of conscientiousness on OPB was mediated by PT, EC, PD, and social self-efficacy. Conscientious individuals are self-disciplined and responsible in interpersonal situations. They are careful of other people’s viewpoints and feelings (PT), and are ready to take actions to maintain interpersonal harmony and reduce inconsistencies (Song and Shi, 2017). These features are conductive to PB/OPB. The positive relationship between EC and conscientious, and negative relationship between PD and conscientious were also found by previous researches (Melchers et al., 2016). Though EC and PD also served as mediators in personality-OPB association, indirect effects of them were relatively weaker than that of PT. Conscientiousness is associated with self-discipline, effort-control, and industry, which are conductive to self-efficacy in social situations (Thoms et al., 1998; Mak and Tran, 2001; Karwowski et al., 2013). These findings suggest that PT and self-efficacy play important roles in linking conscientiousness and OPB. Inconsistent with *Hypothesis 2c*, the mediating effect of MI internalization was not significant. Though conscientiousness is a quasi-moral trait featured by impulse control and restraint (Miller, 2007; Hampson, 2012), its association with MI has not been soundly addressed. Other mediators (e.g., PT, self-efficacy) played stronger roles in the conscientiousness-OPB link than MI internalization.

In consistent with *Hypothesis 2d*, we found that openness can increase OPB via higher levels of PT and social self-efficacy. Openness is an indicator of cognitive ability, which is associated with involvement in community and PB (Aranda and Siyaranamual, 2014; Guo et al., 2019). Song and Shi (2017) found that there was a positive openness-PT association. Openness means sensitivity and insightfulness in understanding others and satisfying their needs (Costa et al., 2013), suggesting that open-minded individuals are better in thinking from the perspectives of others and act accordingly. Furthermore, openness means flexibility and tolerance for ambiguity in interpersonal communications, which are conductive to high social self-efficacy and greater willingness to help others online (Mak and Tran, 2001; Khang and Jeong, 2016). Surprisingly, FS, an empathy component most closely related to openness (Melchers et al., 2016), did not mediate the openness-OPB link. Results showed that the role of FS on OPB were not significant in this study and some researchers even found a negative relationship between FS and prosocial reasoning (Lai et al., 2012). These findings suggested that the effect of FS on prosocial engagement is quite limited. Additionally, MI internalization did not mediate the openness-OPB link. The reason may be that openness is not a core element of moral trait (Miller, 2007).

Consistent with our *Hypothesis 2e*, neuroticism was negatively related to OPB via lower levels of PT, EC, and social self-efficacy. Neurotic individuals are susceptible to negative emotions like anxiety and depression (Lee, 2009), which prevent them from putting themselves in other’s places and caring for others feelings (Mooradian et al., 2011). Neuroticism is characterized by helplessness, poor self-control, and social avoidance, which will lead to lower social self-efficacy (Mak and Tran, 2001; Karimzade and Besharat, 2011), and consequently less prosocial engagement both online and offline. Consistent with Guo et al. (2018b), this study found that neuroticism could increase OPB through elevated PD. Witnessing others’ sufferings are especially painful for neurotic individuals (Mooradian et al., 2011). And lend a helping hand to relieve others’ sufferings is one way to alleviate PD of one own (Song and Shi, 2017). This can be the reason why neuroticism could facilitate PB/OPB via elevated PD.

This is the first study examining the mechanisms through which personality is associated with OPB. We found that personality traits influence OPB in different ways. First, the most important mediator is social self-efficacy, which directly translates personality into actual behavior (Jennings et al., 2015; Khang and Jeong, 2016). Previous literature suggests that morality is not conductive to prosocial engagement if the ability to meet the needs of the sufferers is absent (Sun et al., 2019). Second, another important mediator is PT. Understanding others’ inner states may be the basis of other-oriented responses (Galinsky et al., 2008; Decety and Yoder, 2016). The role of EC is less important because OPB does not require face-to-face interactions which may interfere with emotional perception (Einolf, 2008; Barrett et al., 2011; Konrath et al., 2011). Third, the mediating role of MI is less important than expected. Its mediating effect may have been suppressed by other mediators in the hypothesized models (Daniel et al., 2015; Taguri et al., 2018). Finally, based on the co-construction theory we further propose that the mediating mechanisms between the personality-OPB associations are similar to that in the personality-PB association.

### Limitations and Future Directions

One limitation of this study is that the identification of mediators is not inclusive, leading to the fact that the personality-OPB association was only partially accounted for by these mediators. Other mediators, such as moral judgment (Eisenberg, 2000; Hardy, 2006), interpersonal trust (Deng et al., 2017), empathic self-efficacy (Caprara et al., 2010), should be examined in future studies. The second limitation is that the recipients of OPB have not been distinguished. OPB toward diverse types of recipient (e.g., friends, strangers) may be differently affected by personality (Oda et al., 2014). The third limitation is that the frequency of Internet use (Ryan and Xenos, 2011), which may be an important factor influencing online helping, has not been examined in this study. Additionally, using a OPB measure that has been only validated in Chinese context may limit the generalizability of the findings of this study.

Despite the above limitations, this study provides empirical evidence that personality can consistently affect PBs across different contexts. Besides, personality is stable and difficult to be changed, researchers and caseworkers can consider cultivating individuals’ empathy (especially perspective taking) and social self-efficacy (the belief that one can perform well in social interactions) to effectively promote online PB, so as to enable the helpers and recipients benefit more from the use of the Internet.

## Data Availability Statement

The datasets presented in this study can be found in online repositories. The names of the repository/repositories and accession number(s) can be found below: https://osf.io/p32ye/files/.

## Ethics Statement

The studies involving human participants were reviewed and approved by the Academic Committee at Shandong Normal University. Written informed consent to participate in this study was provided by the participants’ legal guardian/next of kin.

## Author Contributions

JL analyzed the data, wrote the original manuscript, and revised the manuscript. QG designed the work, provided data analysis ideas, and revised manuscript. BM helped revise the manuscript and performed the final proofreading. SZ helped revise the manuscript and provided statistical support for data analyzing. PS collected the data and provided data analysis software. All authors contributed to the article and approved the submitted version.

## Conflict of Interest

The authors declare that the research was conducted in the absence of any commercial or financial relationships that could be construed as a potential conflict of interest.
